# Supplementary material for: Human induced pluripotent stem cell-derived three-dimensional cardiomyocyte tissues ameliorate the rat ischemic myocardium by remodeling the extracellular matrix and cardiac protein phenotype
Source: PLoS One. 2021 Mar 15;16(3):e0245571. doi: 10.1371/journal.pone.0245571 (PMC7959395; doi:10.1371/journal.pone.0245571)
Supplement: Attachment — Submitted filename: revise.docx [file pone.0245571.s002.docx]

February 16, 2021

Emily Chenette, PhD

Deputy Editor-in-Chief

*PLOS ONE*

Dear Dr. Chenette,

I wish to submit the revised version of our manuscript titled “***Human induced pluripotent stem cell-derived three-dimensional cardiomyocyte tissues ameliorate the rat ischemic myocardium by remodeling the extracellular matrix and cardiac protein phenotype*”** (manuscript ID:PONE-D-20-41017) for publication in *PLOS ONE*.

We thank the two reviewers for their time and effort in carefully reviewing our manuscript and providing constructive comments and suggestions to improve it. We have addressed all the comments in the revised manuscript. The changes are marked in red font color and yellow marker. We have also provided point-by-point responses to the reviewers’ comments, attached herewith.

We hope that the revised manuscript is now suitable for publication in your journal.

Thank you for your consideration. I look forward to hearing from you.

Sincerely,

Yoshiki Sawa, MD, PhD

Division of Cardiovascular Surgery, Department of Surgery

Osaka University Graduate School of Medicine

2-2, Yamada-oka, Suita, Osaka 565-0821, Japan

Phone: +81-6-6879-3154

Fax: +81-6-6879-3163

Email: sawa-p@surg1.med.osaka-u.ac.jp

**Reviewer 1:**

Q1.

What was the control used in the hypoxia and in vivo study? Page 9, line 134 the authors have mentioned uncoated, is this the right control to be used to compare the layer-by-layer generated iPSC-CT? Generally, for generation of CT one would use collagen, fibronectin or Matrigel?

A1.

**Response:** Thank you for your comment. The FN/G coated cells and uncoated cells were compared in the hypoxia experimental system.

*In vitro*, when 3 × 10^6^ cells were placed in a 24-well cell culture insert without coating, tissues could not be created as the cells became heterogeneous. Thus, we were not able to compare coated and uncoated 3D-hiPSC-CTs.

Additionally, when 3D-hiPSC-CTs were generated, we used only fibronectin and gelatin—not collagen—as a small population of vimentin-positive cells are found in iPS induction; there is a possibility that collagen derived from fibroblasts may be present.

Q2.

Generally, in case of the cardiomyocytes maintained in media containing FBS, the FBS tend to prevent the beating of cells. Authors need to clarify this, how they were able to get beating cardiomyocytes for the experiment during transplantation?

A2.

**Response:** Thank you for pointing this out. In this study, 3D-iPS-CTs were cultured in a medium containing 10% FBS serum. We confirmed that the cardiomyocytes were beating in this experimental system daily and that the myocardial tissue itself was beating at the time of transplantation. FBS was flushed out avoid immunological rejection prior to CT transplantation. We have included the relevant sentences in the manuscript.

Q3.

What is the conformation test for the coating of fibronectin and gelatin on iPSC-CMs? Authors must perform analytical evaluations similar to SEM and FTIR and provide data to improve rigor of the current study.

A3.

**Response:** We appreciate the helpful suggestion. As suggested, we have presented the TEM image.

We have included the following: “Ultrastructural analysis of 3D-hiPSC-CT using transmission electron microscopy. In the 3D-hiPSC-CT, irregularly shaped hiPSC-CMs aggregate in several layers. Each manufactured cardiomyocyte contained some myofibrils and mitochondria with a few cristae. The cells contacted each other via desmosomes (Fig 5A).

The implanted 3D-hiPS-CT showed clear thickened myofibrils, Z-band, and mitochondria with many lamellar cristae. The adherens junction and desmosome were also disclosed between the iPSC-CM (Fig 5B).”

In regards to the conformation test for coating, the iPS-CM was coated by the method by our co-author Akashi [1], which confirmed coating; thus, we had not confirmed the results of coating again.

[1] Nishiguchi A, Matsusaki M, Miyagawa S, Sawa Y, Akashi M. Dynamic nano-interfaces enable harvesting of functional 3D-engineered tissues. Adv Healthc Mater. 2015;4(8): 1164-1168. doi:[10.1002/adhm.201500065](https://doi.org/10.1002/adhm.201500065), PubMed: [25728509](http://www.ncbi.nlm.nih.gov/pubmed/25728509).

Q4.

There is no sham group in the experiment setup. Authors should include sham group during the analysis of the *in vivo* data.

A4.

**Response:** *In vitro*, when 3 × 10^6^ cells were placed in a 24-well cell culture insert without coating, tissues could not be created as the cells became heterogeneous. Thus, the transplant experiment was a comparative experiment between sham control—which had no transplanted tissue—and the 3D-hiPS CT transplant.

Q5.

Page 6 line 86 replace iPSC-CM as iPSC.

A5.

**Response:** Thank you, as suggested we have revised iPSC-CM to iPSC.

Reviewer2:

**Response:**

Thank you for your peer review despite your busyness. We will continue to strive for the development of the field of regenerative medicine.
